# Topics of the Month

**Published:** 1894-05

**Authors:** 


### TOPICS OF THE MONTH.

A HOSPITAL CAR has been put into service by the Central railroad of New Jersey, which is said to be the first of its kind in the world. It is stationed at Mauch Chunk. The car is divided into two compartments, both of which are fitted up for hospital use. There are cots for the patients, seats, a good supply of medicines and other necessary articles for the care of the injured. The interior is painted a light cream color, which lends a bright and cheerful appearance to the car. Next to providing means for the prevention of accidents, the furnishing of a car specially for the convenience and treatment of those injured is a praiseworthy idea for a railway company to carry out.

IT IS gratifying to know that the Buffalo Health Department has been doing a satisfactory work in reference to sanitary inspections and other methods of enforcing cleanliness. To show how this work increases, it may be remarked that as late as 1891 the number of inspections were less than 1,400, while last year they reached an aggregate of over 18,000. This has become a necessary part of the machinery of the health department, and the Commissioner thinks that it will be equal to all demands in case of a threatened epidemic if he is given proper means to work with. It is unfortunate that there is any "if" in the way of ample financial support to the health department. It should be made responsible for all outbreaks of epidemic diseases, but it cannot be so held unless it is voted ample money by the municipal authorities to meet such outbreaks. Let the Common Council take heed and give Commissioner Wende all the funds necessary for the prosecution of his work.

———

The following group of paragraphs, taken from a recent number of the *Post-Graduate*, possess interest to all who favor a separate State license for the practice of medicine. It is amazing that "learned professors" should be found endeavoring to obstruct the progress making in regard to improved methods of preparation for the practice of medicine:

The *Post-Graduate* thinks that some of the learned professors of the Buffalo Medical College might have been in better business than in instigating, or at least in heeding, the petition of their students for a reduction of the fee for the examination required by the State Board. What a boundless sympathy these Buffalo College doctors have! We do not notice that they have reduced any of their own fees, and, disguise their action as we may, it was simply an attempt to belittle the influence of this beneficent law. Up to this writing, like all their predecessors, who have proposed all kinds of amendments at each session since the law was enacted, they have failed to convince the Legislature and the medical profession at large that the law needs any substantial amendment.

It is written, "A man's foes shall be those of his own household." It seems too bad that the medical profession should be no exception to this rule, but here also that our own family should be in a state of discord. It is beyond endurance that, after these years of struggle to put New York State abreast of the civilized nations of the old world, cultivated and intelligent practitioners of medicine, who happen to be professors in medical colleges, should endeavor, as some of them do, to undo the effects of what is a great advance for the State of New York.

We hope we may encourage the Medical Society of the State of New York to persist in their efforts to defend always the principle involved in this law, so that it shall not fail to continue to be a barrier against the foes that spring up on every hand. Under the old system the medical colleges, nearly always private institutions, were the sole arbiters as to who should constitute a medical practitioner in the State.

It may be as well, first as last, for these medical professors to understand that students are rapidly gaining knowledge as to the value of the medical diplomas that faculties issue. They will soon decline to attend a school that does not properly and adequately prepare them for the State examination.

---

THE advantages of an accurate clinical thermometer are such as to place any such instrument very high in the estimation of physicians. There are so many inferior thermometers in the market that are distributed as prizes or sold at low rates, that the effect often is to bring clinical thermometry into disrepute.

Taylor Brothers Company, of Rochester, N. Y., manufacture a clinical thermometer which is certified as to accuracy, and experience has demonstrated that it has no superior in any market in the world. They reject every imperfect thermometer, which adds much to the cost of production, hence makes its price higher than that charged for inferior instruments. If any physician wishes to obtain an accurate clinical thermometer, he can do so by purchasing a Taylor certified instrument.

---

MONTANA, though one of the youngest of the states, has shown herself worthy of taking an advanced position among her older sisters with reference to separate state examination for license to practise medicine. She is one of the first, if not the very first, to revoke a license where its holder was proven guilty of unprofessional, immoral and dishonorable conduct. The case is entitled, The Montana Board of Medical Examiners *vs.* Dr. E. S. Kellogg, and on appeal to the District court, Judges Hunt and Buck sustained the action of the medical board in revoking Dr. Kellogg's license. We hope the precedent here established will stimulate similar action on the part of medical boards in other states in parallel cases.

---

DR. J. W. LONG, of Richmond, Va., professor of diseases of women and children in the Medical College of Virginia, has recently made a successful hysterectomy for fibrous tumor, complicated by pregnancy. This was done on December 26, 1893. The operation consisted in a long abdominal incision ; no adhesions were found, tumor and uterus were turned out, the ovarian and uterine arteries ligated and cut, and the whole mass cut away at the internal os. The blood supply was thoroughly controlled, hemostatic forceps not even being necessary after severing the pedicle. The pedicle was trimmed, the canal cauterized with carbolic acid, superimposed plains of silk sutures applied, and the stump dropped. The peritoneum was sutured on either side, the pelvic cavity made dry with sponges, and the abdominal wound closed without drainage. She recovered without rise of pulse or temperature, no nausea, pain or tympany.

The specimens were shown at the Richmond Academy of Medicine in January, 1894, and consisted of a group of five or six tumors, together with a pregnant uterus in the fourth month· Pregnancy was diagnosticated beforehand, and the operation was undertaken with a full knowledge of its existence. This is believed to be the first hysterectomy done in the South for fibrous tumor with complicating pregnancy. A full report of this interesting case is published in the *Virginia Medical Monthly* for April, 1894.

---

An epidemic of typhoid fever has occurred at Montclair, N. J., in which up to this writing seventy cases of the disease have been reported to the Health Board, and there have been twelve deaths. It arose from polluted milk sold to some forty or fifty families by a dairyman, in whose family there occurred a case of typhoid fever. The sale of the milk was stopped and the epidemic checked, but the mischief already had been done.

This case strongly emphasizes anew the danger of infection through milk and the urgent need of the strictest watchfulness to insure immunity therefrom.

In Sajou's Annual for 1893 it is related that ten years ago the editor of the *British Medical Journal* showed that, up to date, seventy-one epidemics in England had been traced to milk—namely, fifty of enteric fever, fifteen of scarlet fever, and six of diphtheria, the total number of sufferers being 4,800. Since then a great many milk epidemics have been recorded, probably far more than during any previous period, and there are the strongest reasons for believing that various other diseases, especially including that most dreaded and fatal of all, tuberculosis, are propagated in the same way. Milk is largely consumed without cooking or any process that would destroy noxious bacteria. Nor can it be cleansed and purified in any way by the consumer who wishes to use it raw. Full dependence must, therefore, be placed in its wholesomeness as supplied by the vendor. Hence, the importance of a careful inspection and a rigid system of internal quarantine, under state or national authority, is again pointed out, in the hope that some action may be taken to protect the lives and health of the people.

---

THE second annual report of the Sheppard Asylum, a hospital for mental diseases, located at Towson, Baltimore county, Md., has been published. It is a handsome imperial octavo brochure, beautifully illustrated with photographic reproductions containing views of the buildings and grounds from various points of view, as well as numerous photogravures of interior views. This hospital was built and is supported by the munificent benefaction of the late Moses Sheppard. It is a monument to individual philanthropy and is worthy of imitation by wealthy American citizens. The hospital is presided over by Dr. Edward N. Brush, formerly of Buffalo, who is well known as one of the most celebrated alienists in this country. It is a satisfaction to know that he has attained such conspicuous success as an expert in mental disease, and that he has succeeded in making the Sheppard Asylum one of the best hospitals of its kind, even in the short time during which he has acted as its superintendent.

---

THE twenty-fifth annual report of the New York Physicians' Mutual Aid Association has been published. It is an excellent showing of the workings of the association for the year 1893, and indicates the continued prosperity of this great charity. It is, however, a charity that renders something in return to the families of any of its members who may die during the year. Its assessments are low, and it issues a death certificate of $1,000, and it pays more promptly than any insurance company doing business in this state. Dr. Daniel Lewis, of New York, is president, and Dr. Robert Campbell is treasurer. Application for membership may be made to Dr. W. G. Gregory, 530 Main street, secretary and treasurer of the auxiliary committee of Buffalo.

---

WE ARE indebted to Prof. J. P. Roberts, director of the Cornell University Agricultural Experiment Station, for Bulletin No. 65: a very valuable paper on tuberculosis in relation to animal industry and public health, by Prof. James Law, a copy of which will be sent to physicians and members of boards of health on application. The author first speaks of the prevalence and relative importance of this dread disease, and says that "the average ratio of deaths from tuberculosis to the total mortality is fourteen per cent."

Its prevalence in lower animals is next treated, in which we find that tuberculous cattle number in Bavaria 0.225 per cent.; in France, 0.5 per cent.; in Belgium, 0.4 per cent.; in Paris, 6 per cent.; in Holland, 20 per cent.; in Edinburgh, 26 per cent.; in Pommerania and Bomberg, 50 per cent. The American figures given by the Bureau of Animal Industry are, for Baltimore, from 2.5 to 3.5 per cent.

The author then goes on to speak of the contagiousness of tuberculosis, and gives a brief outline of the germ with some of its characteristics, including its indestructibility. Under the heading of accessory causes of tuberculosis we find hereditary predisposition; close buildings with lack of ventilation; dark stables; insufficient or unwholesome food; overtaxing; breeding too young; ill-health and the like, all carefully treated.

After a careful description of the symptoms, the use of tuberculin as an aid to diagnosis is ably treated. A very strong protest is entered against the use of meat and milk of tuberculous animals for food. It is clearly shown that infection is possible from these sources and that in addition to this, the danger attendant upon their use is very great owing to the introduction of the specific poison of bacillus tuberculosis into the system. Under the heading, danger from milk, we find, "milk is more to be dreaded than meat, because the udder is often the seat of tuberculosis, and the milk is usually taken uncooked." A number of cases are cited to show that the infection of man through milk is possible. In speaking of poisoning by ptomaines and toxins in meat and milk of tuberculous animals, the author concludes as follows: "The germ which might have remained comparatively dormant and harmless in the absence of the poisoned meat and milk is by these stimulated to a more deadly energy." The remainder of the paper is devoted to consideration of how to meet the danger.

---

THE monthly report of the Buffalo Department of Health for March, 1894, contains a record of deaths from enteric or typhoid fever which is a terrible indictment against the person or persons responsible for using the water from the Bird island inlet in the month of February. The record is as follows: enteric fever, 45; enteritis, 4; entero-colitis, 3; gastro-enteritis, 3; total, 55.

Deaths are also reported from diarrhea, 5; dysentery, 2; gastritis, 7; intestinal catarrh, 2; peritonitis, 3; ulceration of intestines, 1.

---

THE Illinois State Board of Health has amended the schedule of requirements for admission to medical colleges, by taking out of the hands of the faculties entrance examinations in the elementary branches, and requiring a certificate of graduation from a literary and scientific college or high school, or a second grade teacher's certificate. This action looking toward higher requirements for entrance to medical colleges is to be commended. The number of recent graduates in medicine who cannot write the English language properly is amazingly too large.

---

THE fortieth annual report of the Superintendent of Public Instruction for the State of New York, for the school year ending July 25, 1893, is chiefly remarkable in that it opposes the present system of two educational departments—namely, the University of the State of New York, governed by Regents, and the Department of Public Instruction, presided over by the author of this report. The curious feature, however, is that, when read between the lines, this profound and erudite superintendent would really prefer to have the University system and the Board of Regents wiped out and his own department made supreme. We are of the opinion that it would be better if a unification of the educational system were brought about, and everything pertaining to the subject merged in the University plan and placed under the control of the Regents. A separate department of public instruction, independently presided over by a superintendent, is quite superfluous.

In the *Literary Digest* for April 21, 1894, is an article condensed from the London *Journal of Education*, entitled, An English View of the University of the State of New York, that deserves to be widely read. After commending the system in liberal terms, it contrasts it with the partisan department of public instruction, and says, further, that under a university law of 1892 good service has been done in closing up discreditable and fraudulent institutions of a quasi-professional type, calculated to do much harm in legal and medical circles. Also, that "in no department is the beneficent action of the university more evident than in the medical." A physician licensed in other states or foreign countries must satisfy the regents of the worth of his credentials before he can practise in the State of New York.

And yet there are "learned professors" who would break down this splendid system.

